# Dual Passivation of Perovskite and SnO_2_ for High‐Efficiency MAPbI_3_ Perovskite Solar Cells

**DOI:** 10.1002/advs.202001466

**Published:** 2021-01-29

**Authors:** Yali Chen, Xuejiao Zuo, Yiyang He, Fang Qian, Shengnan Zuo, Yalan Zhang, Lei Liang, Zuqin Chen, Kui Zhao, Zhike Liu, Jing Gou, Shengzhong (Frank) Liu

**Affiliations:** ^1^ Key Laboratory of Applied Surface and Colloid Chemistry Ministry of Education Shaanxi Key Laboratory for Advanced Energy Devices Shaanxi Engineering Lab for Advanced Energy Technology School of Materials Science and Engineering Shaanxi Normal University Xi'an 710119 China

**Keywords:** europium, perovskite, photovoltaics, solar cells

## Abstract

So far, most techniques for modifying perovskite solar cells (PSCs) focus on either the perovskite or electron transport layer (ETL). For the sake of comprehensively improving device performance, a dual‐functional method of simultaneously passivating trap defects in both the perovskite and ETL films is proposed that utilizes guidable transfer of Eu^3+^ in SnO_2_ to perovskite. Europium ions are distributed throughout the SnO_2_ film during the formation process of SnO_2_, and they can diffuse directionally through the SnO_2_/perovskite interface into the perovskite, while most of the europium ions remain at the interface. Under the synergistic effect of distributed Eu^3+^ in the SnO_2_ and aggregated Eu^3+^ at the interface, the electron mobilities of ETLs are evidently improved. Meanwhile, diffused Eu^3+^ ions passivate the perovskite to reduce trap densities at the grain boundaries, which can dramatically elevate the open‐circuit voltage (*V*_oc_) of PSCs. Finally, the mainly PSCs coated on SnO_2_:Eu^3+^ ETL achieve a power conversion efficiency of 20.14%. Moreover, an unsealed device degrades by only 13% after exposure to ambient atmosphere for 84 days.

## Graphical abstract

A dual‐functional method of simultaneously passivating trap defects in both perovskite and electron transport layer (ETL) films is proposed. Europium ions distribute throughout SnO_2_ film and diffuse into perovskite, while most of Eu^3+^ remain at the interface. Under the synergistic effect of distributed Eu^3+^, the electron mobility of ETL is improved and the trap density of perovskite is also reduced.

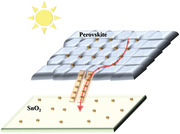

## 1 Introduction

The power conversion efficiency and device lifetime are both key factors for the assessing efficient perovskite solar cells. Recently, the certified power conversion efficiency (PCE) of perovskite solar cells (PSCs) has risen steeply to 23.7%,^[^ ^1^, ^2^, ^3^, ^4^, ^5^, ^6^, ^7^, ^8^, ^9^, ^10^ ^]^ however, in comparison with commercial solar cells, such as crystalline silicon, polycrystalline silicon and Cu(In, Ga)Se_2_ solar cells, the poor device stability of PSCs is still the obstacle to obtaining a market share.^[^ ^11^, ^12^, ^13^ ^]^

Most of the reported methods aiming to improve either or both the PCE and stability of PSCs focus on optimizing the perovskite or electron transport layer (ETL). Regarding perovskite photon absorber layers, their soft crystal lattices tend to easily deform, particularly under various stresses, such as moisture, oxygen, and ultraviolet light exposure, and even under the electric field and thermal stress during device operation.^[^ ^14^, ^15^, ^16^, ^17^ ^]^ Various techniques, such as encapsulation, ultraviolet filtration, and modification have been used to delay the degradation of perovskite materials under the stress of environmental and device operational factors for maintaining long‐term stability of PSCs. Additionally, methods that introduce additives into perovskite films to promote PCE are widely used.^[^ ^18^, ^19^ ^]^ For instance, goethite quantum dots interact with iodine, lead and methylamine, resulting in the retardation of crystallization kinetics to achieve perovskite films with high crystallinity and large grain size;^[^ ^20^ ^]^ Imidazole sulfonate zwitterions are introduced to regulate the crystal orientation of MAPbI_3_ film so it is highly ordered to passivate trap states;^[^ ^21^ ^]^ and the conjugated polymer poly(bithiophene imide) is incorporated within grain boundaries to improve the crystallinity of perovskite film for reducing its defects.^[^ ^22^ ^]^

Besides optimizing the photon absorber, as an important part of a PSC, the ETL must possess high electron mobility to extract photo‐induced carriers because effectively transferring carriers to the external circuitry can promote the PCE of devices. Meanwhile, a suitable ETL should present decent optical transmittance for ensuring enough light reaches the perovskite absorber. Various strategies for optimizing ETLs are reported, such as ethylene diamine tetraacetic acid complexing SnO_2_,^[^ ^23^ ^]^ using [6,6]‐Phenyl C61 butyric acid to modify ZnO,^[^ ^24^ ^]^ or doping TiO_2_ with Sm^3+^ and Eu^3+^ ions.^[^ ^25^ ^]^ Fabricating an ETL with organic chemicals or rare‐earth ions can not only tune the Fermi level of the ETL to better match the conduction band of the perovskite for facilitating charge carrier transfer but also modify the interface between the perovskite and the ETL to induce the perovskite to crystalize with better quality and larger grain size. However, more effective and convenient techniques need to be developed to improve both the perovskite and ETL by simultaneously repairing the different trap defects in the photon absorber and ETL, finally achieving effective photon‐induced charge carrier separation and transfer for higher PCE. This one‐step technique can reduce the cost of PSC engineering by a significant margin.

Europium ions can perform as a redox shuttle to selectively oxidize Pb^0^ and reduce I^0^ defects simultaneously in MAPbI_3_ thin films, and the elimination of both Pb^0^ and I^0^ defects promotes the photovoltaic properties of MAPbI_3_ PSCs with PCE up to 19.67%.^[^ ^10^ ^]^ This MAPbI_3_ film is deposited by a traditional two‐step method. In this process, Eu(acac)_3_ additive is added to the PbI_2_/DMF (dimethylformamide) precursor solution. In addition to modifying the perovskite materials, Eu^3+^ and Sm^3+^ co‐doped TiO_2_ are prepared by the pulsed laser deposition method. The incorporated Eu^3+^ ions in cooperation with Sm^3+^ optimize the TiO_2_ ETL, achieving higher electron extraction and lower interfacial recombination; therefore, power conversion efficiency as high as 19.01% can be obtained for a MAPbI_3_ solar cell.^[^ ^25^ ^]^ Evidently, for the purpose of simultaneously reducing the trap defects in perovskite and ETL films, europium additive is a good choice; however, the above‐discussed preparation methods present restrictive europium oxidation effects or high‐temperature/cost synthesis processes.

We demonstrate a guidable transfer method to achieve Eu^3+^ incorporation in both the ETL and perovskite in one step. Europium and tin ions are simultaneously deposited to form SnO_2_:Eu^3+^ film on FTO (F‐doped SnO_2_) glass. We observed directional diffusion of Eu^3+^ from the SnO_2_ ETL to the MAPbI_3_ perovskite film, which leads to accumulation of large amount of Eu^3+^ at the perovskite/ETL interface. Eu^3+^ ions synergistically eliminate the trap defects in both the ETL and perovskite films, resulting in an improved electron mobility of the SnO_2_ and grain boundary passivation within MAPbI_3_ films. The champion fabricated PSC attains a PCE as high as 20.14%, and, when exposed to the ambient atmosphere, the unsealed PSC presents a slow degradation by only 13% after 84 days. All these results indicate that our dual‐functional technique of europium passivation is extremely effective and convenient.

## 2 Results and Discussion

**Figure 1:**
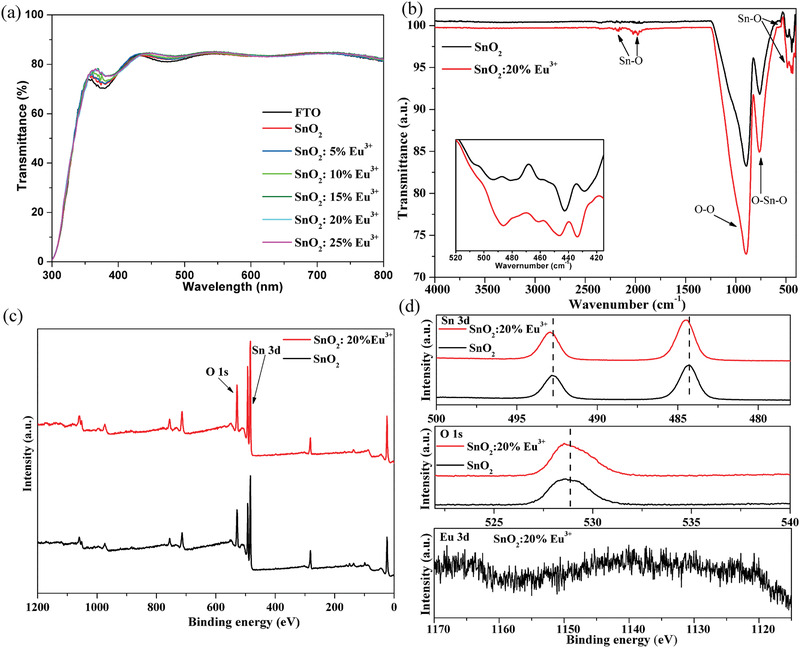
a) Optical transmission spectra of FTO substrates, SnO_2_ and SnO_2_:Eu^3+^ films on FTO substrates. b) Fourier‐transform infrared spectroscopy (FTIR) spectra of SnO_2_ and SnO_2_:20% Eu^3+^ films. c) Typical X‐ray photoelectron spectroscopy (XPS) spectra of SnO_2_ and SnO_2_:20% Eu^3+^ films. d) High‐resolution XPS spectra of Sn 3d, O 1s, and Eu 3d of SnO_2_ and SnO_2_:Eu^3+^ films.

Since the refractive indices of FTO substrates and SnO_2_ films are different, the reflectance can be influenced by the modification of SnO_2_ films. Doping with Y^3+^ ions improves the antireflection ability of the SnO_2_ films and results in an increase in the optical transmittance in the region of 350 to 625 nm for the substrates.^[^ ^26^ ^]^ The optical transmittance spectra of SnO_2_ and SnO_2_:Eu^3+^ films on FTO substrates are shown in **Figure** **1**a. It is interesting to note that doping Eu^3+^ ions can improve the optical transmission properties of FTO/SnO_2_ substrates. In both the 350–410 nm and 440–600 nm regions, the optical transmittances of FTO/SnO_2_ substrates are enhanced with increasing concentration of Eu^3+^ dopant.

The variation of optical transmittance is caused by the reduction of light scattering on the surface of SnO_2_:Eu^3+^ films, which should be related to the variable morphology of the homogeneously distributed SnO_2_:Eu^3+^ grains. Thus, the top‐view scanning electron microscopy (SEM) images of SnO_2_ and SnO_2_:Eu^3+^ films were measured and are shown in Figure S1, Supporting Information. The images show that the SnO_2_ film appears to be flat, uniform and pinhole‐free. After the introduction of Eu^3+^ ions, many nanoparticles appear on the grain surface. Figure S2, Supporting Information shows atomic force microscopy images of SnO_2_ and SnO_2_:Eu^3+^ films deposited on FTO substrates. The calculated data reveal that the root‐mean‐square roughness decreases from 29.9 to 22.1 nm with Eu^3+^ doping. Note that the smoother surface is beneficial to film‐forming of the perovskite layer.^[^ ^23^ ^]^

FTIR spectra is used to study the interaction between the dopant Eu^3+^and matrix SnO_2_. As shown in Figure 1b, the peaks around 760 cm^−1^ belong to the O—Sn—O symmetric stretch, and the peaks at 895 cm^−1^ are attributed to the O—O stretching about vibration of the oxygen adsorbed on the surface of SnO_2_ films. All the weaker peaks at ≈2180, ≈2027, and ≈1977 cm^−1^ are due to Sn—O stretching vibrations.^[^ ^23^, ^27^ ^]^ All of these absorption peaks are unaffected by Eu^3+^ doping; however, for the SnO_2_:20% Eu^3+^ sample, the characteristic asymmetric stretching peaks on the SnO_2_ surface shift to 486, 446, and 434 cm^−1^,^[^ ^27^ ^]^ which demonstrates that Eu^3+^ ions might enter into the SnO_2_ crystal lattice and affect the SnO_2_ surface.

To further clarify the interaction between Eu^3+^ and SnO_2_, the XPS spectra for SnO_2_ and SnO_2_:20% Eu^3+^ films are measured and shown in Figure 1c. Clearly, the two Sn peaks and O peak are centered at ≈484, ≈493, and ≈529 eV, respectively. Meanwhile, high‐resolution Sn 3d, O 1s, and Eu 3d spectra are displayed in Figure 1d. In comparison with pristine SnO_2_ films, the shifts of ≈ 0.2 and 0.05 eV of the Sn 3d and O 1s peaks, respectively, can be observed in the SnO_2_:20% Eu^3+^ films, indicating that the Eu^3+^ dopant affects the SnO_2_ surface. Furthermore, the presence of trivalent Eu^3+^ can be confirmed by the observed binding energies at ≈1167.2 and 1139.8 eV, which are attributed to the 3d_3/2_ and 3d_5/2_ orbitals of Eu^3+^.

**Figure 2:**
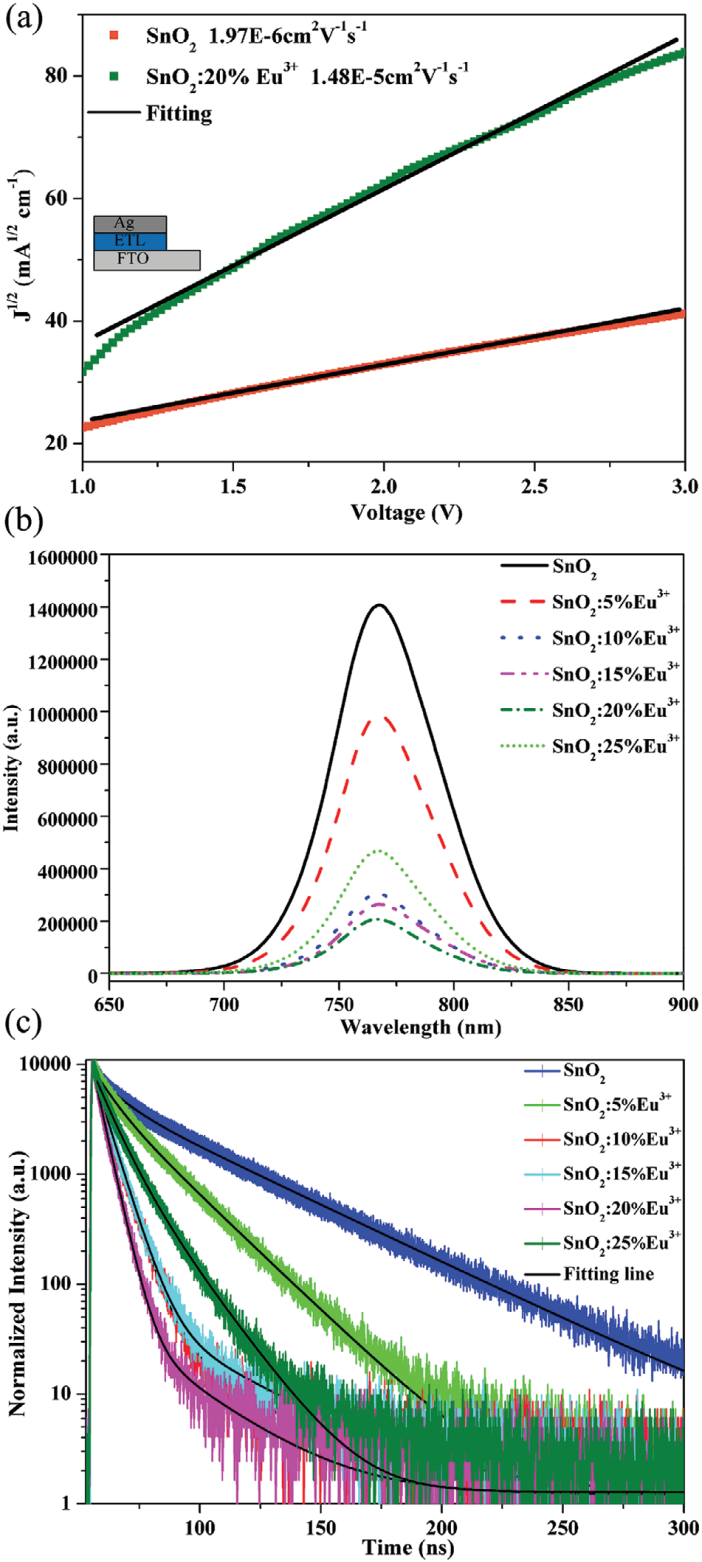
a) Electron mobility for SnO_2_ and SnO_2_:20% Eu^3+^ films; the inset shows the device structure of FTO/ETL/Ag. b) Steady‐state photoluminescence (PL) and c) time‐resolved photoluminescence (TRPL) spectra of perovskite films deposited on different ETL substrates.

The electrical properties of semi‐conductive films can be characterized by the Hall Effect measurements, such as conductivity type, resistivity, mobility and carrier concentration. The average Hall coefficient, resistivity, mobility, and carrier concentration of SnO_2_ and SnO_2_:20% Eu^3+^ were measured and shown in Table S1, Supporting Information, respectively. The average Hall coefficients indicate that SnO_2_ and SnO_2_:20% Eu^3+^ are both well ETL films. Meanwhile the recorded resistivities, mobilities, and carrier concentrations prove Eu^3+^ doping can reduce resistivity and promote mobility in SnO_2_ films. It is known that the electron mobility of ETLs is the key factor for the performance improvement of PSCs. The various ETLs are also measured by the space charge‐limited current method,^[^ ^23^ ^]^ and the results shown in **Figure** **2**a. It is found that the electron mobility of SnO_2_:20% Eu^3+^ is 1.48 × 10^−5^ cm^2^ V^−1^ s^−1^, which is about eight larger than that of SnO_2_ (1.97 × 10^−6^ cm^2^ V^−1^ s^−1^). High electron mobility can effectively promote electron transfer in an ETL, thus the charge consumption at the interface between the ETL and perovskite is reduced, finally resulting in improved efficiency of PSCs.

The PL spectra of the perovskite deposited on different ETL substrates are presented in Figure 2b. Compared with the FTO/SnO_2_/perovskite sample, significant PL quenching is observed with increasing Eu^3+^ concentration in the samples on SnO_2_:Eu^3+^ ETL substrates. The data demonstrate optimized SnO_2_:20% Eu^3+^ presents the most appealing merits with the highest electron mobility. Figure 2c displays the normalized TRPL for perovskites on various ETLs. The lifetimes and corresponding proportions are listed in Table S2, Supporting Information. The lifetime decay curves have two parts: a slow decay component *τ*_1_ and a fast decay component *τ*_2_. Generally, *τ*_1_ is attributed to the radiative recombination of free carriers captured by the traps in bulk materials and *τ*_2_ originates from the quenching of charge carriers in the transportation process through the interfaces.^[^ ^23^, ^28^ ^]^ The FTO/SnO_2_/perovskite sample presents a long *τ*_2_ lifetime of 37.55 ns, but it quickly decreases with increasing Eu^3+^ in SnO_2_ films. Smaller *τ*_2_ dominating PL decay indicates that electrons can be effectively extracted from the perovskite layer to the ETL with minimal recombination loss at the interface. In the sample of perovskite deposited on SnO_2_:20% Eu^3+^, *τ*_1_ was increased slightly to 7.65 ns, but *τ*_2_ was considerably shortened to 3.53 ns; moreover, the lifetime contributions of *τ*_1_ and *τ*_2_ are 13.05% and 86.95%, respectively. These results indicate that the Eu^3+^ dopant in SnO_2_:20% Eu^3+^ film leads to suppressed charge carrier recombination at the interfaces, which remarkably dominates the overall charge carrier transport process. Moreover, sharply decreased *τ*_2_ indicates that the carrier transfer efficiency at the interfaces can be significantly promoted by doping Eu^3+^ into SnO_2_. To summarize, the reduced loss of charge carriers at the interfaces drastically improves the carrier transfer efficiency in SnO_2_:20% Eu^3+^, which can be considered as a potential electron extraction layer for planar‐type PSCs.

**Figure 3:**
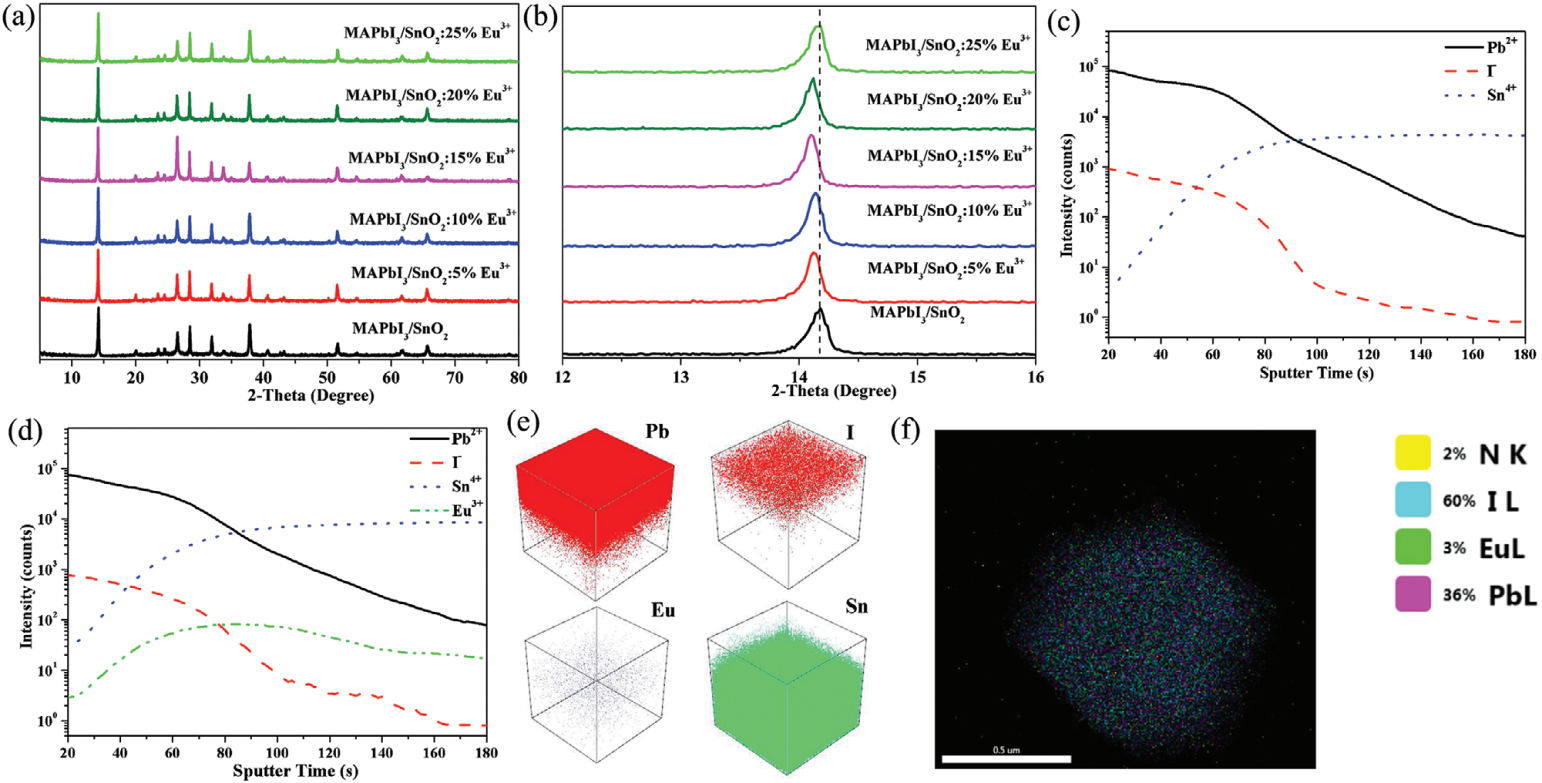
a) XRD patterns and b) enlarged XRD patterns of MAPbI_3_ on SnO_2_ and SnO_2_:Eu^3+^ ETL substrates. Secondary ion mass spectroscopy (SIMS) of c) perovskite/SnO_2_ and d) perovskite/SnO_2_:20% Eu^3+^ films. e) The spatial distribution of Pb, I, Eu, and Sn in perovskite/SnO_2_:Eu^3+^ films. f) Energy dispersivespectroscopy (EDS) mapping image of perovskite/SnO_2_:Eu^3+^ film.

The doped europium ions in MAPbI_3_ perovskite films can simultaneously reduce Pb^0^ and I^0^ defects for achieving high PCE, and they more easily concentrate at surfaces and grain boundaries or intercalate into two adjacent lattices.^[^ ^10^ ^]^ In our research, X‐ray diffraction (XRD) patterns of MAPbI_3_ on various ETL substrates (SnO_2_ and SnO_2_:Eu^3+^) in **Figure** **3**a,b indicate that europium ions can spread from SnO_2_:Eu^3+^ films into MAPbI_3_ perovskite films. When the europium ion concentration in SnO_2_ films is adjusted from 5 to 20 mol%, an obvious shift of the MAPbI_3_ (110) diffraction peak is observed. Because the radii of Eu^3+^ and Pb^2+^ are 94.7 and 119 pm, respectively, the shifts of the diffraction peak indicate that Eu^3+^ may prefer to intercalate into two adjacent lattices than to enter into the perovskite crystal lattice, in accordance with the reported density functional theory result.^[^ ^10^ ^]^ When the doping concentration of Eu^3+^ exceeds 20%, excess Eu^3+^ might enter Pb^2+^ sites of the perovskite crystal lattice. Therefore, the shift of diffraction peak would reverse, and the corresponding photovoltaic properties of PSCs would be affected.

The SIMS curves shown in Figure 3c,d present the distribution of Pb, I, Sn, and Eu along the depth direction of perovskite/SnO_2_ and perovskite/SnO_2_:20% Eu^3+^ films, respectively, and their spatial distribution images are presented in Figure 3e. In the crossing regions of the SIMS profiles, sharply varying Pb, I, and Sn contents means those regions are the interfaces between the perovskite and SnO_2_ (or SnO_2_:Eu^3+^) films. It is observed that Eu^3+^ ions are evenly distributed in the SnO_2_ film, but they tend to aggregate at the interface between the perovskite and SnO_2_:20% Eu^3+^ films, and subsequently, through the process of diffusion, a few Eu^3+^ ions enter the perovskite film. Therefore, the shifts of the asymmetric stretching Sn—O peaks in the FTIR spectra (Figure 1b) and the dramatically increased proportions of *τ*_2_ lifetime in the TRPL spectra (Figure 2c) are observed, which are both related to the aggregation of Eu^3+^ at the interface between the perovskite and SnO_2_:Eu^3+^ films. An EDS mapping image of perovskite/SnO_2_:Eu^3+^ film is presented in Figure 3f. Although the amount of Eu^3+^ ions pervading the perovskite film is very low, their distribution is uniform throughout the perovskite film.

The surface coverage of perovskite films is also very important for high‐performance PSCs.^[^ ^27^, ^28^, ^29^ ^]^ The aggregation of Eu^3+^ on the top interface of SnO_2_:Eu^3+^ films may affect the nucleation and growth of perovskite films. A smaller contact angle can result in a reduced Gibbs free energy facilitating heterogeneous nucleation; meanwhile, the formation of more crystal nuclei will accelerate the process of thin film growth from nuclei to island structures, then to networked, and finally into a continuous film.^[^ ^23^, ^30^, ^31^ ^]^ The contact angles of SnO_2_ and SnO_2_:Eu^3+^ films are measured and presented in Figure S3, Supporting Information. All calculated contact angles indicate that increased concentration of dopant Eu^3+^ in SnO_2_:Eu^3+^ films reduces the contact angle on the surface, and moreover, the contact angle is a minimum of 10.2° on the surface of SnO_2_:20% Eu^3+^ films, which can result in lower surface energy and accelerated perovskite crystallization during the growth of the networked structure.^[^ ^30^, ^32^ ^]^ However, the grain sizes of perovskite films are not obviously affected by the different substrates, as shown in the SEMs of Figure S4, Supporting Information.

Grain boundary plays a critical role in determining the charge collection efficiency and stability of PSCs. The passivation of grain boundary can reduce the trap densities of perovskite films to improve the performance of PSCs. Different grain boundary passivation methods are being studied, such as introducing the PbI_2_‐rich phase at grain boundaries in MAPbI_3_ PSCs,^[^ ^33^ ^]^ or using carbon quantum dots additive to passivate the uncoordinated lead ions on grain boundaries of MAPbI_3_ PSCs.^[^ ^34^ ^]^ In our work, the aggregated Eu^3+^ at the grain boundaries and the interfaces can reduce Pb^0^ and I^0^ defects further to passivate grain boundaries. Thus the trap densities of perovskites on different ETLs are evaluated by the space charge‐limited current measurements of electron‐only devices fabricated with the structure ITO (indium tin oxide)/ETL/perovskite/PCBM (phenyl‐C61‐butyric acid methyl ester)/Ag, and the corresponding dark current–voltage (*I*–*V*) curves are shown in Figure S5, Supporting Information. At low bias voltage, the linear correlation shown as red lines indicates an ohmic response. When the bias voltage increases above the kink point, the current suddenly increases with a nonlinear correlation (cyan line), which reveals that the traps in the perovskite film are totally filled. The bias voltage corresponding to the kink point between the linear and nonlinear correlation is defined as the trap‐filled limit voltage (*V*_TFL_). The trap densities (*N*_t_) of the perovskites on different ETLs are calculated by the equation *N*_t_ = (2*V*_TFL_ *εε*_0_)/(*eL* ^2^),^[^ ^35^, ^36^ ^]^ where *ε* and *ε*_0_ are the relative dielectric constant for MAPbI_3_ perovskite and vacuum permittivity, respectively, *e* is the electron charge and *L* is the thickness of the MAPbI_3_ perovskite film. It is obvious that the trap densities of the perovskites were reduced from 1.69 × 10^16^ to 1.31 × 10^16^ cm^−3^ by increasing the amount of Eu^3+^ dopant to 20% in the SnO_2_ substrate, and this reduction is attributed to the passivation of grain boundaries caused by increased Eu^3+^ at grain boundaries and interfaces.

**Figure 4:**
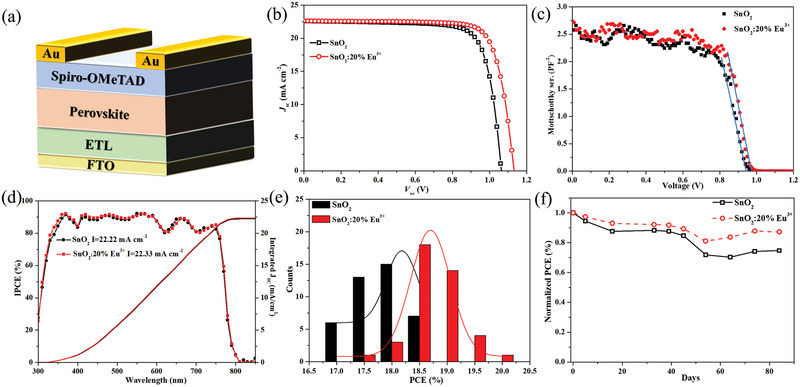
a) The designed device configuration. b) Current density−voltage (*J–V*) curves, c) Mott–Schottky plots and d) The incident‐photon‐to‐charge conversion efficiency (IPCE) of the PSCs with different ETL substrates. The integrated current densities from the IPCE curves were recorded under the AM1.5G spectrum. e) The PCE distribution of the PSCs based on the different ETLs. f) Long‐term stability test for planar‐type PSC devices with different ETLs without any encapsulation under ambient conditions (35% humidity in the dark).

According to the above discussion, SnO_2_:Eu^3+^ is expected to be a better ETL for PSCs than the pristine SnO_2_ film. Therefore, planar‐type PSCs with different SnO_2_ films as ETL substrates are designed with the structure shown in **Figure** **4**a. MAPbI_3_ and Spiro‐OMeTAD are used as the photon absorber layer and hole‐transport layer, respectively. The thicknesses of the perovskites are nearly unchanged by doping 20% Eu^3+^ into SnO_2_ film, as shown in the cross‐sectional SEM images of PSCs in Figure S6, Supporting Information.

Figure 4b and Figure S7, Supporting Information present the *J*–*V* curves of PSCs on various ETL substrates, and the corresponding key parameters, such as the open‐circuit voltage (*V*_oc_), the short‐circuit current (*J*_sc_), fill factor (FF), and PCE are all summarized in Table S3, Supporting Information. The maximum PCE of the devices based on the SnO_2_ ETL substrate is 18.66%, with the corresponding *V*_oc_ = 1.06 V, *J*_sc_ = 22.57 mA cm^−2^, and FF = 77.77%. It is exciting that the optimal PCE can be increased to 20.14% by changing the SnO_2_ ETL to SnO_2_:20% Eu^3+^ ETL, with corresponding *V*_oc_ and FF dramatically increased to 1.13 V and 78.76%, respectively. The increased FF are due to the reduced trap density in perovskite film; however, the improved *V*_oc_ should be attributed to the reduced energy loss in MAPbI_3_ PSCs.^[^ ^37^ ^]^

Mott–Schottky analysis based on capacitance–voltage (*C*–*V*) measurement can be used to investigate the change of built‐in electric field and is carried out to further understand the charge carrier trapping and accumulating behaviors with the incorporation of Eu^3+^ dopant.^[^ ^38^, ^39^, ^40^, ^41^ ^]^ Figure 4c presents the *C* ^−2^–*V* plots for MAPbI_3_ perovskite PSCs, and the corresponding values of the built‐in electric fields (*V*_bi_) in the PSCs based on different ETLs are obtained from the Mott–Schottky equation *C* ^−2^ = (2(*V*_bi_ − *V*))/(*A* ^2^ *eɛɛ*_0_ *N*), where *C* is the capacitance under applied voltage, *V*_bi_ is the built‐in potential, *V* is the applied bias, *A* is the device area, *ɛ* is the relative permittivity, *ɛ*_0_ is the vacuum permittivity, and *N* is the free carrier concentration at the edge of the depletion layer.^[^ ^42^, ^43^ ^]^ The *V*_bi_ for the PSC coated on SnO_2_:20% Eu^3+^ ETL is 0.972 V, which is larger than 0.942 V of the control device without europium dopant. The enhanced built‐in potential provides more driving force to separate the photogenerated charge carriers, resulting in an extended depletion region, which suppresses electron‐hole recombination, finally contributing to the increase of *V*_oc_.

On the other hand, the *V*_oc_ is also determined by the quasi‐Fermi level separation of electrons and holes in the light‐dependent dynamic equilibrium condition, and the reduced charge carrier recombination and fewer traps within the bandgap can narrow the distribution of defect states and elevate the quasi‐Fermi levels of electrons.^[^ ^44^ ^]^ As shown in Figure S5, Supporting Information, the reduced trap density from 1.69 × 10^16^ to 1.31 × 10^16^ cm^−3^ can be obtained by coating MAPbI_3_ perovskite film on SnO_2_:20% Eu^3+^ substrate. The significantly reduced trap‐assisted recombination elevates the quasi‐Fermi level of electrons, and thus greater quasi‐Fermi level separation can be obtained. As a result, the PSCs fabricated on the SnO_2_:20%Eu^3+^ substrate exhibit a higher *V*_oc_ compared to the control PSCs on the SnO_2_ substrate.

Electrical impedance spectroscopy is carried out to monitor the transfer resistance in PSCs. The Nyquist plots of the PSCs fabricated on different SnO_2_ or SnO_2_:Eu^3+^ films recorded at *V*_oc_ under dark conditions are shown in Figure S8, Supporting Information, and the corresponding equivalent circuit is shown in Figure S9, Supporting Information. It is known that the recombination resistance (*R*_rec_) is in the low‐frequency range,^[^ ^42^ ^]^ and, in our fabricated PSCs, it increases with increased Eu^3+^ doping in the SnO_2_ films. Compared to the control PSCs, the device fabricated on SnO_2_:20% Eu^3+^ substrate shows the largest *R*_rec_ of 289 Ω, which can effectively inhibit charge recombination at grain boundaries and the interface, because the aggregated Eu^3+^ at grain boundaries and the interface decrease the amount of negative defects Pb^0^ and I^0^.

Figure 4d shows the IPCE spectra and the integrated *J*_sc_ values versus wavelength for the PSCs based on different ETLs. The effect on IPCE of Eu^3+^ doping in the ETL is divided into two parts: 1) In the UV region of 300–370 nm, the IPCE intensity is enhanced by Eu^3+^ doping due to the *f–f* transitions absorption of Eu^3+^.^[^ ^45^, ^46^, ^47^, ^48^, ^49^ ^]^ The absorption of Eu^3+^ reduces the damage to the perovskite film from UV light, resulting in the obvious increase of the IPCE; 2) The enhancement of IPCE in the region of 370–550 nm is attributed to the increased transmittance of ITO/SnO_2_:20% Eu^3+^ substrate. The highest observed IPCE value reaches 92%. Meanwhile, the integrated *J*_sc_ increases from 22.22 to 22.33 mA cm^−2^ by using SnO_2_:20% Eu^3+^ film as the ETL substrate, which indicates SnO_2_:20% Eu^3+^ is an excellent ETL for application in PSCs. The stabilized power output of the device with SnO_2_:20% Eu^3+^ ETL is shown in Figure S10, Supporting Information. While maintaining an external bias near the maximum power output point (0.88 V), the stabilized photocurrent for the PSC with SnO_2_:20% Eu^3+^ ETL is 21.37 mA cm^−2^. The results indicate that SnO_2_:20% Eu^3+^ ETL is beneficial to the illumination stability of the MAPbI_3_ perovskite device.

Stability and repeatability are also very important characteristics for PSCs. The PCE distribution histograms for devices with different ETLs are presented in Figure 4e. The device based on SnO_2_:20% Eu^3+^ substrate exhibits excellent repeatability in contrast to that based on the pristine SnO_2_ substrate. Figure 4f shows normalized PCE of the different device exposed to an ambient atmosphere (≈35% humidity) during 84 days in the dark. It is clear that the device based on SnO_2_:20% Eu^3+^ substrate maintains 87% of its initial PCE on the 84th day, but the device coated on SnO_2_ substrate has decreased to 75% of its initial PCE under the same storage conditions. Their stabilities at higher temperature in N_2_ atmosphere, even under continuous illumination and higher humidity are both investigated and shown in Figure S11a,b, Supporting Information, respectively. After storing devices in dark with N_2_ atmosphere at 80 °C for 500 min, the PCE of control device has dropped to 30% of its initial value. But the decrease can be retarded to be very slow by 20% Eu^3+^ doped in SnO_2_, thus the device coated on SnO_2_:20% Eu^3+^ substrate just decreases to 65% of its initial PCE under the same storage conditions. Even under continuous 100 mW cm^−2^ illumination and 40–50% humidity at 60 °C for 300 min, the SnO_2_:20% Eu/Perovskite device can still keep 50% of its initial PCE, but the PCE of SnO_2_/Perovskite device has drop to 40% of its initial value. The comparison demonstrates that the PSCs coated on SnO_2_:20% Eu^3+^ show more excellent stability, which is due to the europium ions can effectively passivate the defects at the grain boundaries and interfaces preventing moisture permeation, further resulting in the improved environmental stability.^[^ ^50^ ^]^

## 3 Conclusion

This work describes a novel dual‐functional method to simultaneously optimize charge transport characteristics of the perovskite and ETL layers which account for the enhancement of performance of the corresponding PSCs. An effective SnO_2_:Eu^3+^ ETL is developed, and the champion device incorporating it achieves a PCE of 20.14%, showing excellent stability by maintaining 87% of its initial efficiency after storage in ambient atmosphere for 84 days. The excellent performance of the PSCs is attributed to the dual‐passivation effect of europium ions in SnO_2_. The uniformly distributed europium dopants reduce the trap defects in the SnO_2_ film, resulting in increased electron mobility of the ETL. The aggregation of europium ions at the interface between the perovskite and SnO_2_ films is beneficial for improving electron transport through the interface by reducing the charge accumulation at the interface. Moreover, the aggregated europium ions passivate the perovskite by reducing the trap density in the grain boundaries, which is favorable to the *V*_oc_ and FF of PSCs. Meanwhile, the aggregated europium ions both on the grain boundaries and interface suppress perovskite degradation by preventing moisture permeation. Our dual‐functional method provides a promising direction toward simultaneously optimizing the ETL and perovskite films, and we believe that the present work will facilitate the development of perovskite photovoltaics.

## 4 Experimental Section

##### Materials

SnCl_2_·2H_2_O (98%) was purchased from Macklin. Thioglycolic acid was purchased from Sigma‐Aldrich. EuCl_3_·6H_2_O (99.99%) was purchased from CIVI‐CHEM. PbI_2_ (99.9985%) was purchased from Alfa Aesar. MAI (99.5%) was purchased from Xi'an Polymer Light Technology Corp. Urea, 4‐tert‐butylpyridine (TBP) and bis(trifluoromethane) sulfonamide lithium salt (Li‐TFSI) were purchased from Aladdin. HCl (37 wt%) and chlorobenzene (≥99.0%) were purchased from Sinopharm Chemical Reagent Corporation Co., Ltd. Spiro‐OMeTAD (≥99.0%) was ordered from Youxuan Tech. 4‐Hydroxybutanoic acid lactone (GBL) and dimethyl sulfoxide (DMSO) were ordered from Alfa‐Aesar.

##### Device Fabrication

The FTO‐coated glass (2.5 × 2.5 cm) was cleaned by sequential sonication in acetone, isopropanol, and ethanol for 30 min each time and then dried under air flow and treated by ozone plasma for 6 min. The undoped and Eu‐doped SnO_2_ layers were prepared by the chemical bath deposition method. Briefly, for the preparation of undoped SnO_2_ ETL, 0.5 g of urea was dissolved in 40 mL deionized water, and then 10 mL thioglycolic acid and 500 mL HCl were added to the aqueous solution. Finally, SnCl_2_·2H_2_O was dissolved in the solution at 0.002 m concentration followed by stirring for 2 min. The clean FTO substrates were immersed in the aqueous solution at 70 °C for 3 h, followed by rinsing in a deionized water sonication bath for 2 min. Then they were dried with flowing air and heat‐treated for 1 h at 180 °C in air. For the preparation of (5, 10, 15, 20, and 25 mol%) Eu‐doped SnO_2_ ETLs, EuCl_3_⋅6H_2_O was added directly to the prepared aqueous solution before immersing the cleaned FTO substrates.

The MAPbI_3_ perovskite solution (1.4 m) was comprised of MAI and PbI_2_ in 1 mL of GBL/DMSO = 7:3 (v/v). The solution was stirred at room temperature for 12 h. Then the FTO/ETL substrates were treated by ozone plasma for 6 min. The solution was spin‐coated onto the FTO/ETL substrate by a consecutive two‐step process at 1000 rpm for 10 s and followed by 3000 rpm for 40 s. During the second step, 200 mL of chlorobenzene was dropped onto the substrate. The films were then annealed at 100 °C for 10 min in a nitrogen‐filled glovebox. 90 mg mL^−1^ spiro‐OMeTAD in 1 mL chlorobenzene with the addition of 36 mL TBP and 22 mL Li‐TFSI solution (520 mg in 1 mL acetonitrile) was spin‐coated onto the perovskite films at 5000 rpm for 40 s. The samples were kept in a desiccator overnight. Finally, 80 nm gold electrodes were deposited on the top of each cell by a thermal evaporator.

##### Device Characterization

XRD spectra were obtained using a D/MAX 2400 diffractometer with Cu Ka radiation (Rigaku). Transmittance spectra were acquired on a PerkinElmer UV‐Lambda 950 instrument. PL (excitation at 510 nm, front‐side excitation) and TRPL spectra (excitation at 510 nm and emission at 768 nm, front‐side excitation) were measured with a PicoQuant FT‐300. Water contact angles were measured using a DataPhysics OCA 20. The surface morphologies of the perovskite films and the SnO_2_ films were characterized by SEM (FE‐SEM; SU‐8020, Hitachi) at an acceleration voltage of 5 kV. XPS measurements were carried out by using a photoelectron spectrometer (ESCALAB 250Xi, Thermo Fisher Scientific). SIMS curves were recorded by the time of flight secondary ion mass spectrometry (TOF SIMS IV, ION TOF GmbH). The *J*−*V* performance of the perovskite solar cells was analyzed using a Keithley 2400 Source Meter under ambient conditions at room temperature, and the illumination intensity was 100 mW cm^−2^ (AM 1.5G Oriel solar simulator) with scan rate 0.2 V s^−1^. The device area of 0.09 cm^2^ was defined by a metal aperture to avoid light scattering from the metal electrode into the device during the measurement. TheIPCE was characterized on a QTest Station 2000ADI system (Crowntech Inc., USA), and the light source was a 300 W xenon lamp. The monochromatic light intensity for the IPCE measurement was calibrated with a reference silicon photodiode. The Hall Effect measurements were recorded by the Hall Effect Measurement System (HMS‐3000).

## Conflict of Interest

The authors declare no conflict of interest.

## Supporting information

Supporting Information
