# Supplementary material for: Dual Passivation of Perovskite and SnO2 for High‐Efficiency MAPbI3 Perovskite Solar Cells
Source: Adv Sci (Weinh). 2021 Jan 29;8(5):2001466. doi: 10.1002/advs.202001466 (PMC7927604; doi:10.1002/advs.202001466)
Supplement: Supplementary file 1 — Supporting Information [file ADVS-8-2001466-s001.pdf]

© 2020 Wiley-VCH GmbH

Supporting Information

for *Adv. Sci.,* DOI: 10.1002/advs.202001466

Dual Passivation of Perovskite and SnO_2 for High-efficiency $MAPbI_3$ Perovskite Solar Cells

Yali Chen, Xuejiao Zuo, Yiyang He, Fang Qian, Shengnan Zuo, Yalan Zhang, Lei Liang, Zuqin Chen, Kui Zhao, Zhike Liu, Jing Gou, and Shengzhong (Frank) Liu**

Supporting Information

Dual Passivation of perovskite and SnO_2 for high-efficiency $MAPbI_3$ perovskite solar cells

Yali Chen, Xuejiao Zuo, Yiyang He, Fang Qian, Shengnan Zuo, Yalan Zhang, Lei Liang, Zuqin Chen, Kui Zhao, Zhike Liu, Jing Gou, and Shengzhong (Frank) Liu**

Y. Chen, X. Zuo, Y. He, F. Qian, S. Zuo, Y. Zhang, L. Liang, Z. Chen, Prof. K. Zhao, Prof. Z. Liu, Prof. J. Gou, Prof. S. (F.) Liu

Key Laboratory of Applied Surface and Colloid Chemistry
Ministry of Education
Shaanxi Key Laboratory for Advanced Energy Devices
Shaanxi Engineering Lab for Advanced Energy Technology
School of Materials Science and Engineering
Shaanxi Normal University
Xi'an 710119, China.
E-mail: goujing@snnu.edu.cn; liusz@snnu.edu.cn

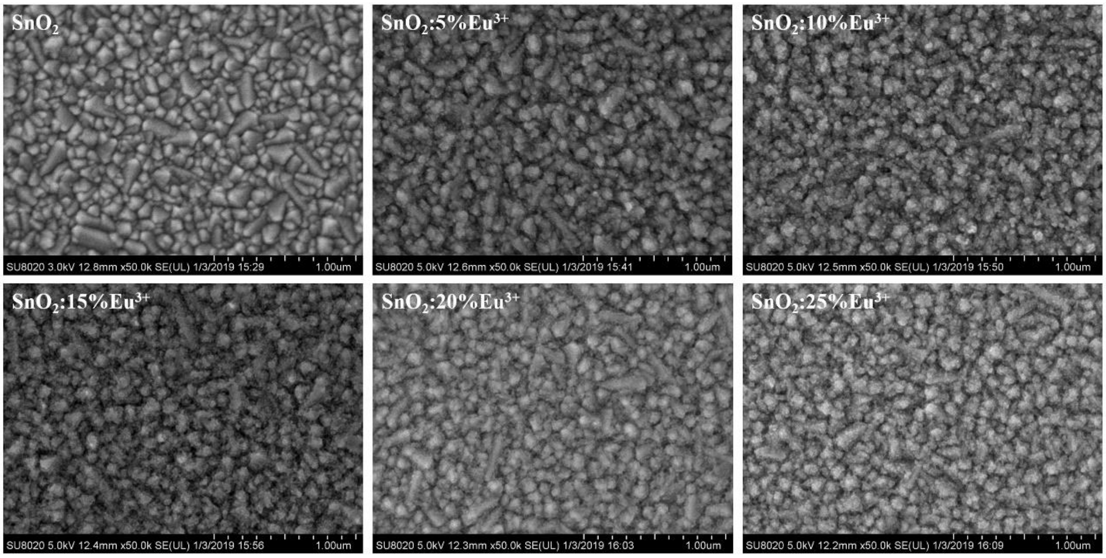

Fig. S1 The SEM images of SnO_2 and $SnO_2:Eu^{3+}$ films.

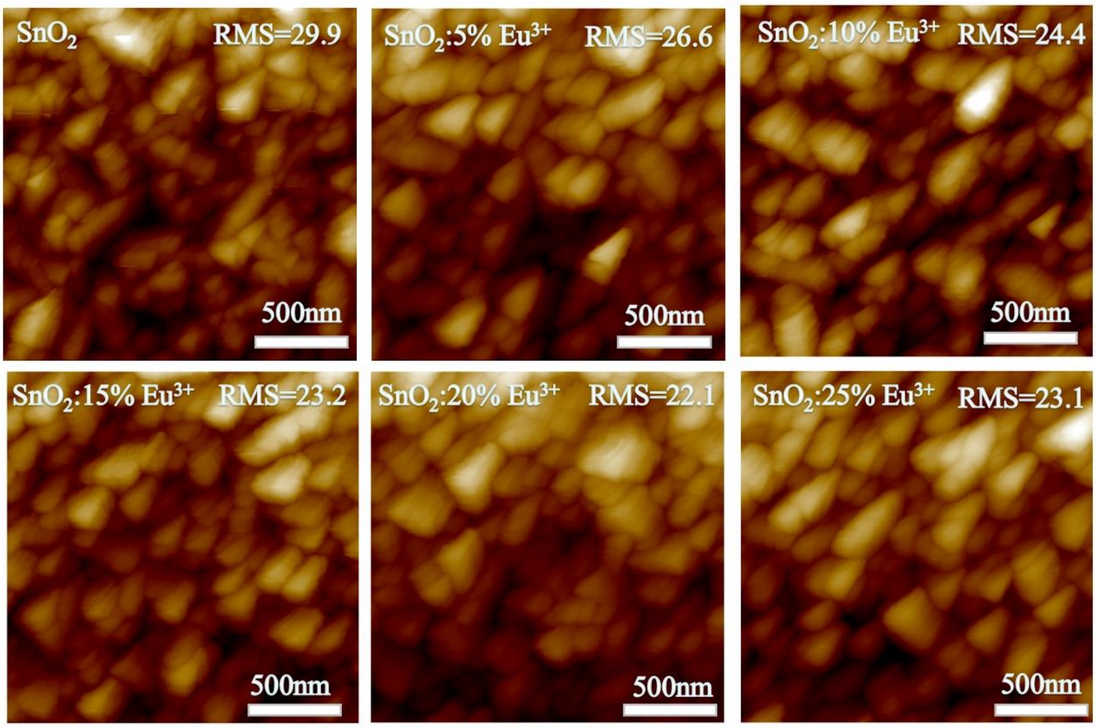

Fig. S2 Atomic force microscopy (AFM) images of SnO_2 and SnO_2:Eu^{3+} films deposited on FTO substrates

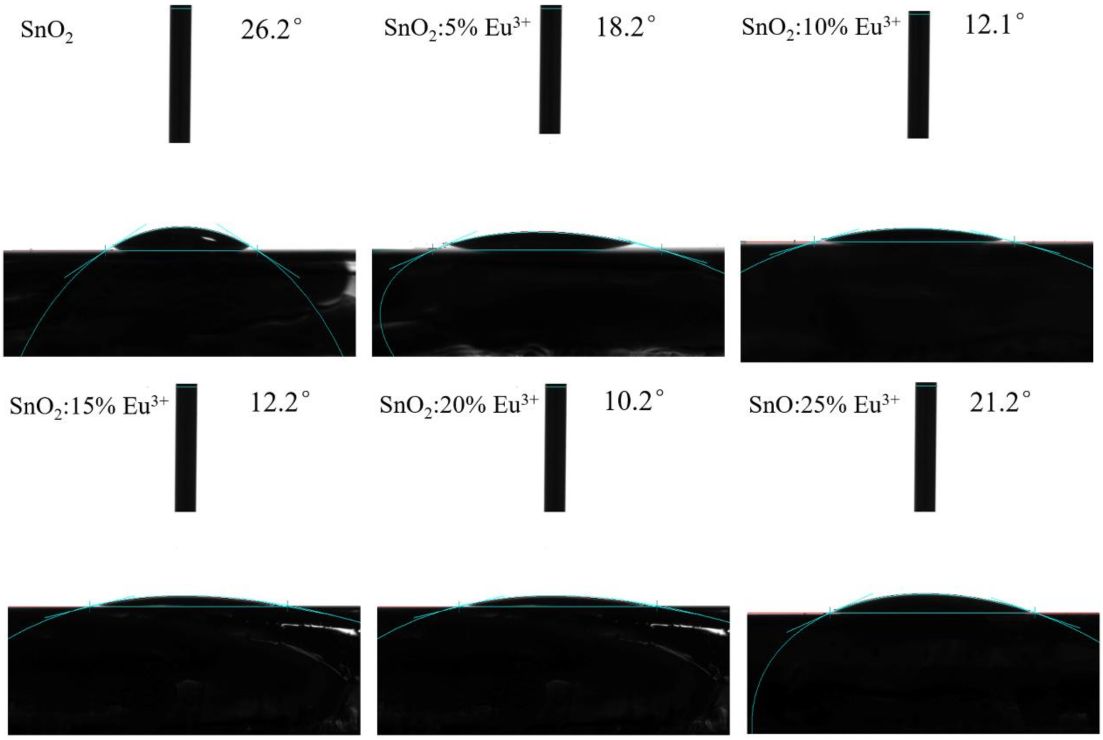

Fig. S3 The contact angles of SnO_2 and SnO_2:Eu^{3+} films.

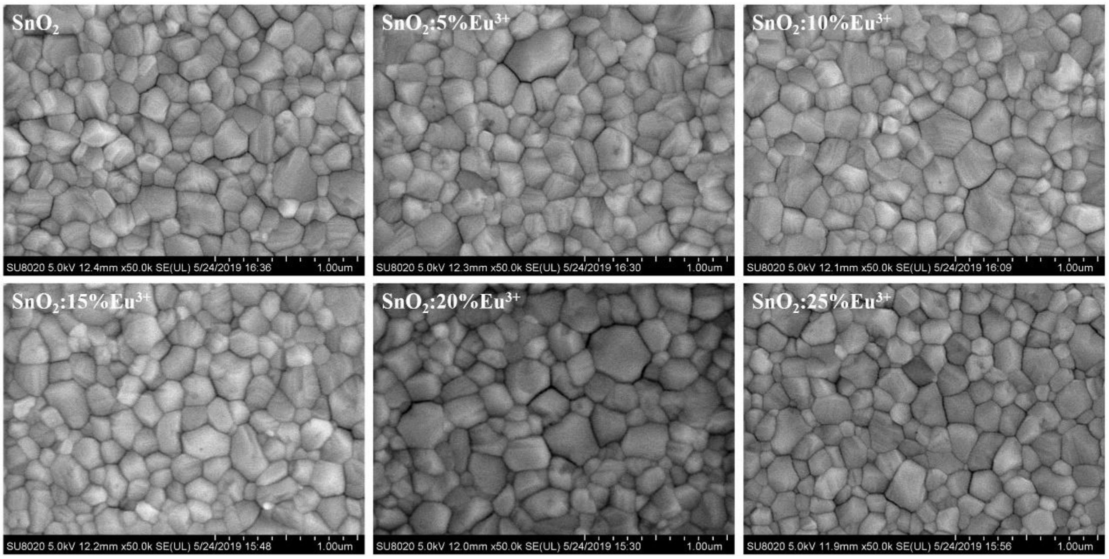

Fig. S4 The SEM images of perovskite films coated on SnO_2 and SnO_2:Eu^{3+} films.

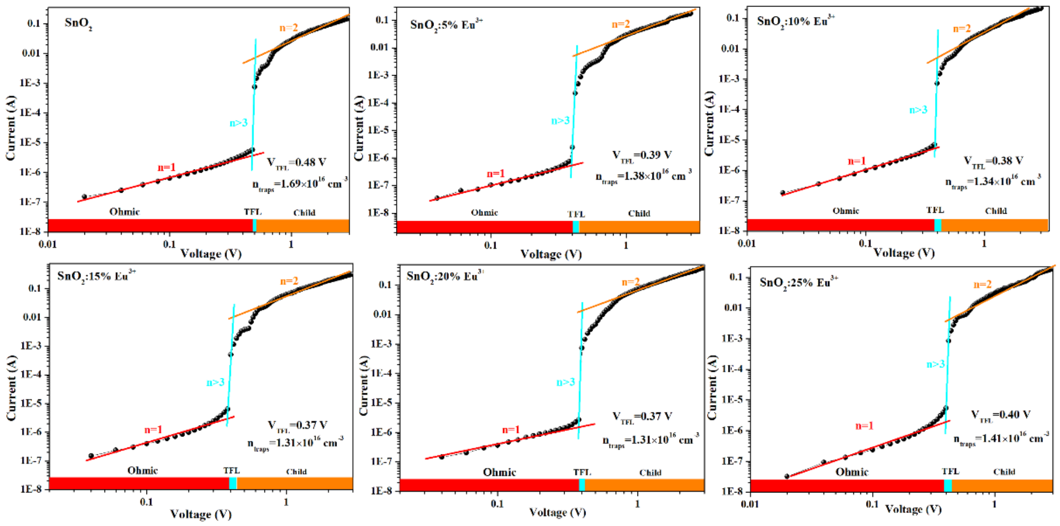

Fig. S5 Dark *I-V* curves of the electron-only devices with different ETL

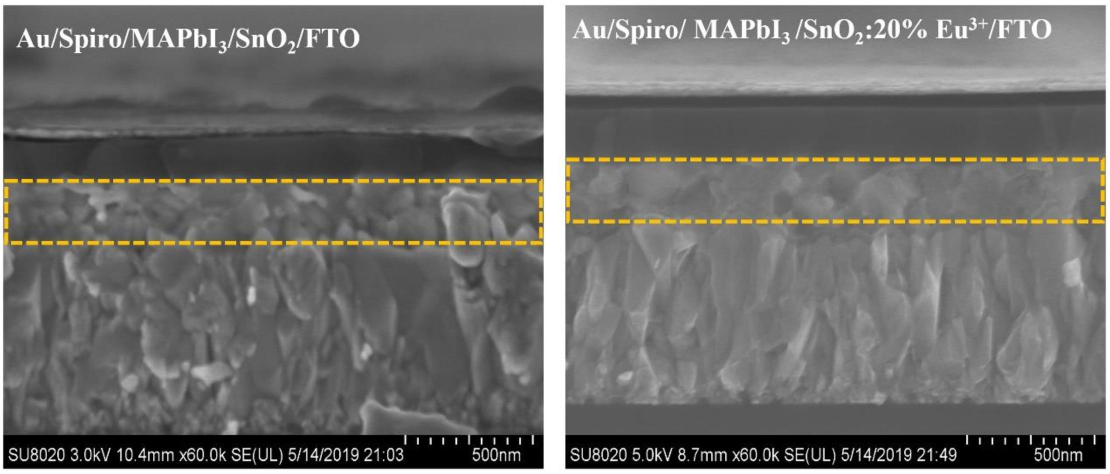

Fig. S6 The cross-sectional SEM images of PSCs with different ETL.

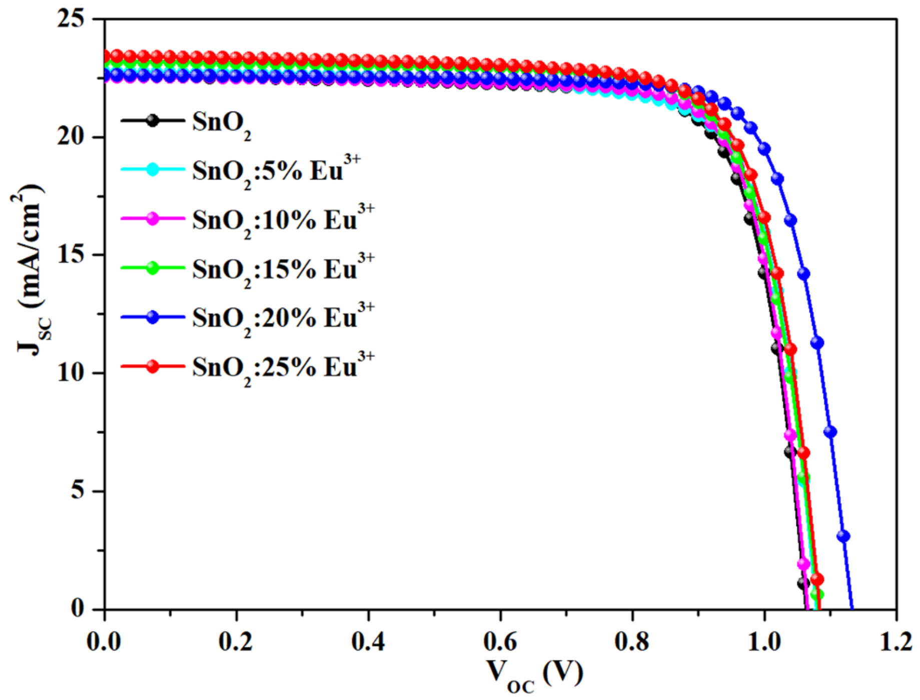

Fig.S7 *J*-*V* curves of the PSCs with different ETLs substrates.

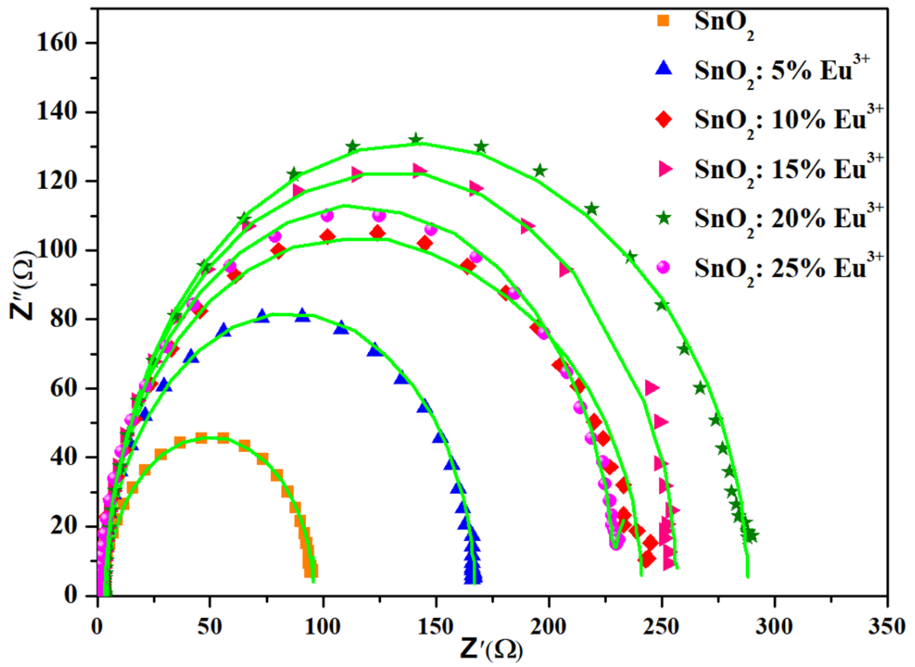

Fig.S8 EIS of planar-type PSCs with various ETLs.

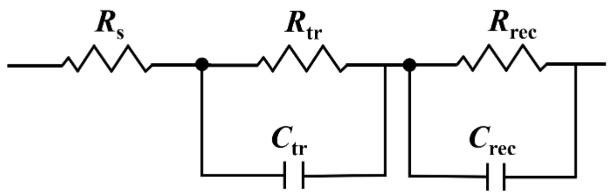

Fig.S9 Equivalent circuit in PSCs.

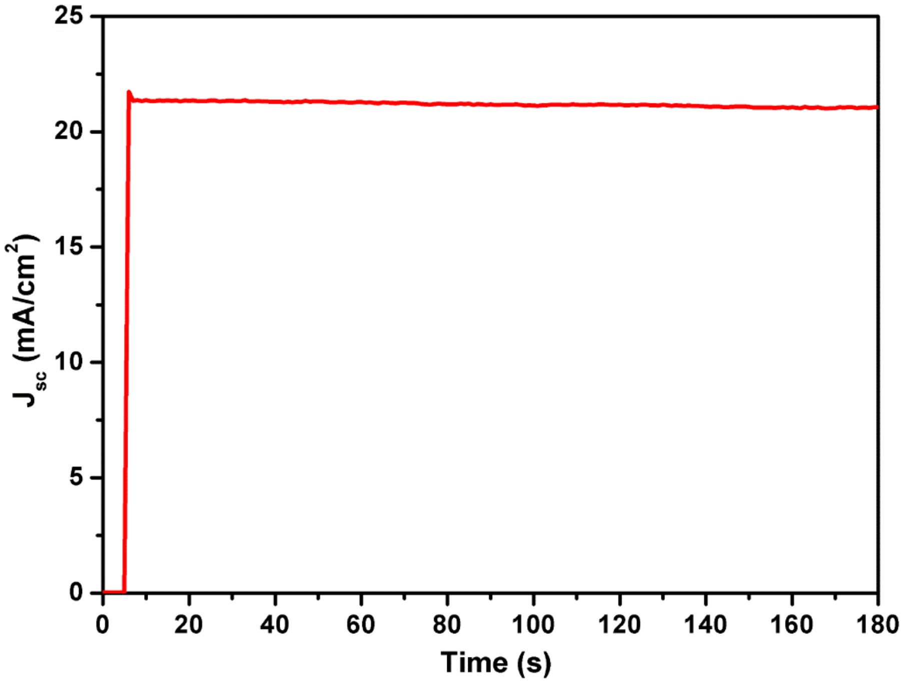

Fig.S10 Stable output curves of current densities of the device with $SnO_2:Eu^{3+}$ at the maximum power point.

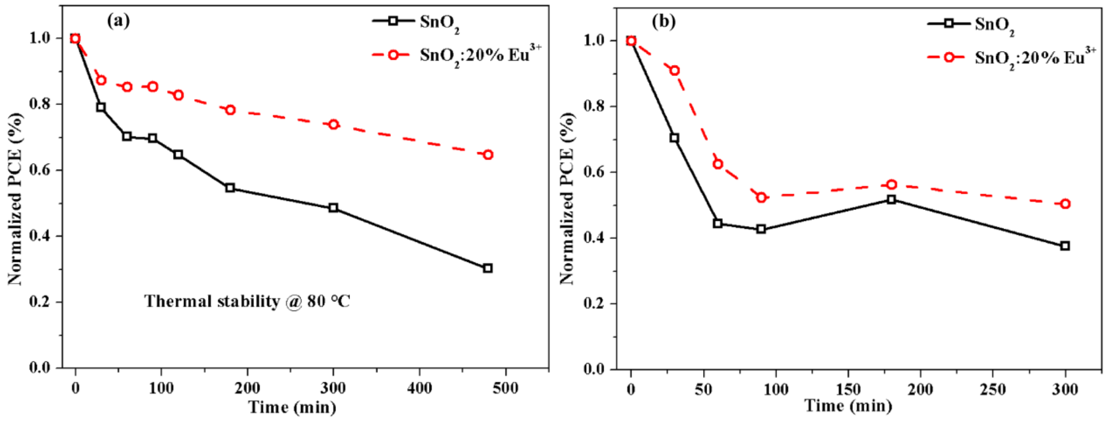

Fig.S11 Stability test for planar-type PSC devices with different ETLs without any encapsulation (a) at 80 °C in dry N_2 atmosphere; (b) under 100 mW cm^{-2} illumination and 40-50% humidity at 60 °C.

Supplementary Table 1 The average Hall coefficient, resistivity, mobility and carrier concentration of SnO_2 and SnO_2:20% Eu films.

Sample	Average Hall coefficient (cm^3 C^{-1})	Resistivity (Ω·cm)	Mobility (cm^2 V^{-1} s^{-1})	Carrier concentration (cm^{-3})
SnO_2	-2.59×10^5	6.24×10^2	4.14×10^2	-2.41×10^{13}
SnO_2:20% Eu	-3.18×10^5	3.36×10^2	9.44×10^2	-1.97×10^{13}

Supplementary Table 2 Parameters of the TRPL spectra of perovskite films deposited on different substrates.

Sample	τ_{ave} (ns)	τ_1 (ns)	% of τ_1	τ_2 (ns)	% of τ_2
FTO/SnO_2/Perovskite	35.20	4.38	39.5%	37.55	60.5%
FTO/SnO_2:5% Eu/Perovskite	17.45	3.94	33.08%	18.85	66.9%
FTO/SnO_2:10% Eu/Perovskite	6.07	2.93	43.59%	7.07	56.41%
FTO/SnO_2:15% Eu/Perovskite	6.16	2.76	38.71%	7.01	61.29%
FTO/SnO_2:20% Eu/Perovskite	4.54	7.65	13.05%	3.53	86.95%
FTO/SnO_2:25% Eu/Perovskite	9.85	3.04	36.32%	10.93	63.68%

Supplementary Table 3 Parameters of PSCs deposited on different substrates.

**The amount of Eu^{3+} in SnO_2:Eu^{3+} (mol%)**	**V_{oc} (V)**	**J_{sc} (mA cm^{-2})**	**FF**	**PCE (%)**
0	1.06	22.57	77.77	18.66
5	1.07	22.75	76.73	18.84
10	1.10	22.41	76.77	19.08
15	1.10	22.25	78.76	19.28
20	1.13	22.61	78.76	20.14
25	1.08	23.41	76.68	19.46
